# Isokinetic testing of quadriceps function in COPD: feasibility, responsiveness, and minimal important differences in patients undergoing pulmonary rehabilitation

**DOI:** 10.1016/j.bjpt.2022.100451

**Published:** 2022-10-17

**Authors:** Anouk A.F. Stoffels, Roy Meys, Hieronymus W.H. van Hees, Frits M.E. Franssen, Bram van den Borst, Alex J. van ’t Hul, Peter H. Klijn, Anouk W. Vaes, Jana De Brandt, Chris Burtin, Martijn A. Spruit, BASES-consortium

**Affiliations:** a Department of Pulmonary Diseases, Radboud University Medical Center, Nijmegen, the Netherlands; b Department of Research and Development, Ciro, Horn, the Netherlands; c Nutrim School of Nutrition and Translational Research in Metabolism, Faculty of Health, Medicine and Life Sciences, Maastricht University, Maastricht, the Netherlands; d Department of Respiratory Medicine, Maastricht University Medical Centre (MUMC+), Maastricht, the Netherlands; e Department of Pulmonology, Merem Pulmonary Rehabilitation Centre, Hilversum, the Netherlands; f Department of Pulmonary Medicine, Amsterdam UMC, Amsterdam, the Netherlands; g REVAL–Rehabilitation Research Center, BIOMED–Biomedical Research Institute, Faculty of Rehabilitation Sciences, Hasselt University, Diepenbeek, Belgium

**Keywords:** Minimal important difference, Muscles, Quadriceps, Rehabilitation, Chronic obstructive pulmonary disease

## Abstract

**Background:**

Isokinetic testing of peripheral muscle function is valid and reliable in patients with chronic obstructive pulmonary disease (COPD).

**Objective:**

To evaluate whether and to what extent isokinetic testing of quadriceps function meets pre-defined test criteria in patients with COPD; to determine the response to pulmonary rehabilitation (PR), and to calculate minimal important differences (MIDs) of isokinetic quadriceps function.

**Methods:**

Retrospective analysis of 2033 patients with COPD (age: 65±9 years, body mass index: 26±6 kg/m^2^, FEV_1_: 49±22% predicted) who followed a comprehensive PR program. Pre and post PR isokinetic quadriceps function was assessed with 30 maximal extension-flexion contractions at an angular speed of 90°/s on a computerized dynamometer. The chosen anchors were 6-min walk test and COPD assessment test.

**Results:**

Pre PR, 27% of the patients performed the isokinetic test incorrectly. In male and female patients with a correct pre and post PR isokinetic test, peak torque (Δ=10±13 Nm or 9% and Δ=7±9 Nm or 10%, respectively) and total work (Δ=263±270 J or 14% and Δ=198±190 J or 15%, respectively) improved significantly. There was no change in work fatigue index following PR. Using distribution-based calculations, MID estimates for peak torque and total work ranged between 6–7 Nm and 97–135 J in males and between 4–5 Nm and 62–99 J in females.

**Conclusions:**

Based on the current test criteria, three in four patients with COPD performed the isokinetic quadriceps test correctly during baseline PR assessment. Furthermore, peak torque and total work, but not work fatigue index, were responsive to PR and sex-specific MIDs were established.

## Highlights

• Evaluation of isokinetic quadriceps testing in COPD is needed to assess its efficacy.
• Isokinetic testing was performed incorrectly in a quarter of patients with COPD.
• Quadriceps peak torque and total work improved following pulmonary rehabilitation.
• Minimal important differences for peak torque and total work were determined.

## Introduction

Peripheral muscle dysfunction is a prominent component of physical impairment and disability^1^ and has been associated with impaired health status, increased utilization of healthcare resources, and mortality in patients with chronic obstructive pulmonary disease (COPD).^2^, ^3^, ^4^ Besides muscle strength, it seems reasonable to evaluate additional muscular features to obtain a more comprehensive overview of skeletal muscle function. One of these aspects is muscle endurance, which represents the muscle's ability to sustain a given task over time.^5^ Quadriceps endurance is more impaired in patients with COPD than quadriceps strength.^6^^,^^7^ This impairment cannot be predicted based on the degree of airflow limitation or maximal muscle strength^8^ and seems to be more closely related to exercise capacity and daily activities than muscle strength.^9^^,^^10^ In addition, recent studies reported significant correlations between muscle endurance and muscle oxidative profile.^11^^,^^12^ This provides a strong rationale for an in-depth assessment of quadriceps endurance in patients with COPD.

Isokinetic, isometric, and isotonic approaches are available to volitionally assess quadriceps endurance in patients with COPD,^2^^,^^7^^,^^13^ amongst which isokinetic testing is most common.^13^ Advantages of isokinetic methods are the dynamic evaluation of muscle function while controlling for angular velocities, amplitude, and duration of movement, and its high reliability.^14^^,^^15^ This latter especially applies when using a computerized dynamometer, provided that three pre-defined criteria of correct test performance are met: completion of all (mostly 30) repetitions, peak torque reached within the first five repetitions, and presence of work fatigue.^15^^,^^16^ However, in patients with COPD, these test criteria have only been validated in relatively small studies and it remains unclear whether and to what extent these criteria are met in clinical settings. In addition, differences in clinical characteristics between patients with and without a correct test performance are unknown.

Pulmonary rehabilitation (PR) is an effective therapy for patients with COPD.^17^ But studies regarding the responsiveness of isokinetic quadriceps endurance and minimal important differences (MIDs) of isokinetic quadriceps strength and endurance following PR are still lacking. Hence, an extensive evaluation regarding isokinetic quadriceps test performance in a PR center is essential.^18^

Therefore, the main objectives of the current study were: 1) to evaluate whether and to what extent the isokinetic testing of quadriceps function meets the pre-defined test criteria (i.e. feasibility) in patients with COPD assessed pre and post PR; 2) to assess differences in clinical characteristics between patients with a correct and incorrect isokinetic test performance; 3) to determine the response to PR and calculate MIDs of isokinetic quadriceps function.

## Methods

A retrospective analysis was performed on pseudonymized clinical data of 3152 patients with a diagnosis of COPD.^19^ These patients were referred for a comprehensive PR program by a chest physician in Ciro (Horn, the Netherlands)^20^ between June 2013 and August 2019. Data during the PR program were systematically collected as part of standard care. At the start of the PR program, patients received a brochure entitled ‘Privacy regulations Ciro.’ One of the paragraphs stated that their information could be used (pseudonymized) for scientific research and statistics and that signing informed consent was not necessary. However, patients had the opportunity to fill in a form when they objected. The information of patients who did not object was entered into a large database.

For this study, individuals younger than 40 years of age were excluded and only data from the first PR program were used when participating on multiple occasions over time. The medical ethical committee informed the authors that the Medical Research Involving Human Subjects Act (WMO) does not apply to this retrospective study and approved the use of data for this study (METC 2019-1384). The Board of Directors of Ciro approved the use of pseudonymized patient records.

### Isokinetic quadriceps testing

**Fig. 1:**
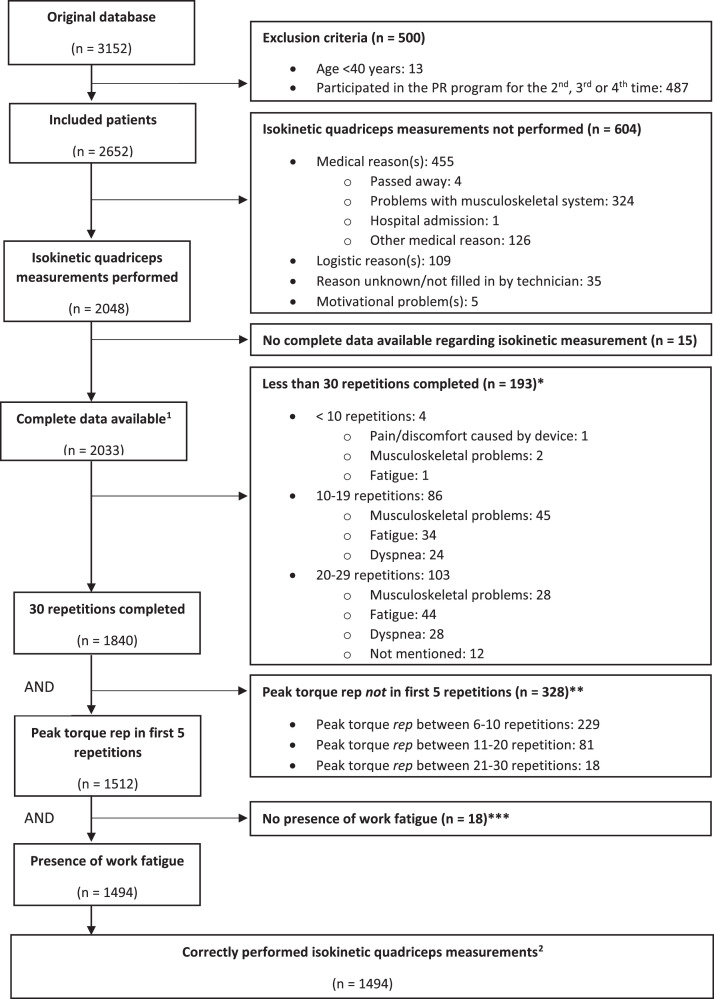
Flowchart of patients who performed the baseline isokinetic quadriceps measurement correctly according to the following three criteria: 30 repetitions completed, peak torque within first 5 repetitions, and presence of work fatigue. *Some patients reported multiple reasons for early test termination. **30 patients with the highest peak torque not in the first 5 repetitions were already excluded due to the fact that they performed less than 30 repetitions. ***50 patients with no presence of work fatigue were already excluded due to the fact that their peak torque was not within first 5 repetitions. ^1^ indicates the group of patients used to evaluate the feasibility of isokinetic test performance at baseline. ^2^ indicates the group of patients used to evaluate the feasibility of isokinetic test performance post PR (Fig 2, Supplementary materials).

The isokinetic quadriceps tests were performed on a computerized dynamometer (System 3, Biodex Inc., Shirley, NY, USA) at baseline (pre PR) and post PR. The patients were positioned on the dynamometer chair with hip and knee placed at a 90° angle and the lever arm attached as described by Frykholm et al.^16^ Extraneous movement during the test was reduced with the use of straps across the chest, waist, thigh, and ankle of the tested leg, and range of motion (ROM) was full knee extension to 90° flexion. Furthermore, participants were instructed to keep their arms crossed over their chest. A warm-up protocol was used in which the patient performed five isokinetic contractions with a progressively higher force production, followed by two minutes of rest. The isokinetic protocol consisted of 30 contractions at an angular velocity of 90°/s with maximal effort during extension and passive (submaximal) flexion. Strong verbal encouragement was provided during all repetitions.^16^ The main outcomes were: peak torque in newton-meter (Nm) and as percentage of predicted values (based on reference values of Borges et al.^21^), total work of all completed repetitions reported in Joules (J), and work fatigue index_10_ and index_5_ as percentages ($\frac{\text{work}\;\text{first}\;5\;\text{or}\;10\;\text{repetitions}\;-\;\text{work}\;\text{last}\;5\;\text{or}\;10\;\text{repetitions}}{\text{work}\;\text{first}\;5\;\text{or}\;10\;\text{repetitions}})*100\%$, respectively; Fig. 1, Supplementary material).^15^ The following criteria for correct execution of isokinetic quadriceps testing were applied: completion of all 30 repetitions, peak torque within the first five repetitions, and presence of work fatigue (positive work fatigue index_10_).^15^^,^^16^

### Other assessments

All clinical characteristics were evaluated during a comprehensive baseline assessment at the start of PR. Demographic data consisted of age, sex, body mass index (BMI), smoking status (pack-years), and use of long-term oxygen therapy. Additionally, pulmonary function was determined using spirometry, static lung volume measurement, and transfer factor for carbon monoxide (MasterScreen PFT/Body; Jaeger, Würzburg, Germany) following the European Respiratory Society (ERS) guidelines.^22^^,^^23^ Disease severity was classified according to the Global Initiative for Chronic Obstructive Lung Disease (GOLD) criteria^19^ and modified Medical Research Council dyspnea scores were obtained with a cut-off value of ≥2 to classify patients as highly symptomatic.^24^ Dual-energy x-ray absorptiometry (Lunar iDXA; DEXAtech Benelux BV, Ridderkerk, the Netherlands) was performed to assess fat-free mass and to calculate the fat-free mass index (fat-free mass divided by height squared).^25^ Symptoms of anxiety and depression were determined using the Hospital Anxiety and Depression Scale with a cutoff value of ≥10 points.^26^ Respiratory muscle strength was obtained using maximal inspiratory and expiratory pressures.^27^ The constant work rate cycle test was performed on an ergometer (Ergoselect; Ergoline, Bitz, Germany) at 75% of the maximal workload (obtained by an incremental cardiopulmonary exercise test^28^) to determine exercise tolerance.^29^ Isotonic peripheral muscle strength was evaluated using 1-repetition maximum leg press and leg extension on standard training apparatus.

Exercise capacity (6-minute walk distance; 6MWD) and health status (COPD Assessment Test; CAT) were determined at baseline and post PR. The 6MWD was performed in duplicate at baseline and once during post PR assessment, following the American Thoracic Society (ATS) guidelines^30^^,^^31^ and using the reference values from Troosters et al.^32^ on a 30-m course.^33^ A threshold of ≥18 points was used for the CAT^34^ to identify patients as highly symptomatic.

### Pulmonary rehabilitation

Patients followed a comprehensive inpatient PR program at Ciro (Horn, the Netherlands) or outpatient PR program within the CIRO+ rehabilitation network based on the latest PR statement of ATS/ERS.^20^ The patient-tailored program consisted of 40 sessions and was supervised by an interdisciplinary team consisting of a chest physician, respiratory nurses, dieticians, occupational therapists, physical therapists, psychologists, and social workers. The cornerstone of the PR program was physical exercise training including resistance training, aerobic training, flexibility exercises, unsupported arm exercises, and daily supervised 30-minute outdoor walks. Additionally, occupational therapy, guidance in medication use and adherence, psychosocial counseling, nutritional advice, education, and/or exacerbation management were provided to the patients if indicated.

Resistance training was performed once per day to improve peripheral muscle strength. Patients performed three to four exercises per training focusing on either upper or lower extremity (varying per day). The initial load of the exercises was individually set at 60% of 1-repetition maximum and was aimed to increase progressively by 3–5% each week. A total of four sets with eight repetitions were performed for each exercise with a 2-min rest period between sets.

Aerobic training included one morning session of treadmill walking and one afternoon session of stationary cycling (or reversed). The initial intensity of walking and cycling was individually based on 6MWD and maximum workload obtained during the incremental cardiopulmonary exercise test, respectively. Borg scores for dyspnea and fatigue (target score of 4–6) were used to make weekly adjustments to the intensity. The type of aerobic training sessions were either (intensive or extensive) interval session or endurance/recovery (both performed once per day). The intensity, duration, rest period, and progression varied for each of these types and are described in the supplementary material (Table 1, Supplementary material). Patients who were unable to perform aerobic training received lower-limb high-frequency neuromuscular electrical stimulation (NMES) twice per day. If patients could only perform one aerobic training session per day, they received one interval training and one NMES session per day.

**Table 1:** Differences between patients with a correct and incorrect isokinetic quadriceps measurement, stratified for sex.

	Male patients		Female patients	
	Incorrect	Correct	Incorrect	Correct
Number	312	745	227	749
Age, years	66 ± 8	67 ± 8	63 ± 9	63 ± 8
BMI, kg/m^2^	26.7 ± 5.4	26.5 ± 5.2	26.1 ± 7.3	25.6 ± 6.4
FFMI, kg/m^2^	17.8 ± 2.2	17.8 ± 2.2	15.4 ± 2.1	15.3 ± 2.0
FEV_1_, L	1.48 ± 0.73	1.49 ± 0.71	1.06 ± 0.52	1.05 ± 0.50
FEV_1_, % predicted	49 ± 23	50 ± 22	49 ± 22	47 ± 21
FEV_1_/FVC, %	41 ± 16	40 ± 15	42 ± 15	39 ± 13§
RV/TLC, %	51 ± 12	49 ± 11§	56 ± 12	55 ± 11
TL_CO_, % predicted	50 ± 19	52 ± 18	49 ± 15	48 ± 16
Smoking, packs per year	47 ± 25	45 ± 25	41 ± 21	40 ± 22
LTOT, n (%)	62 (20)	132 (18)	50 (22)	160 (22)
GOLD (1/2/3/4), %	11/34/31/24	11/31/37/20	11/29/39/21	8/30/40/22
GOLD (A/B/C/D), %	8/29/7/56	11/26/9/54	5/18/5/72	6/23/8/63
mMRC ≥ 2, n (%)	266 (86)	594 (81)§	203 (90)	643 (86)
CAT total ≥ 18 points, n (%)	205 (71)	490 (70)	172 (79)	553 (77)
HADS-Anxiety ≥ 10 points, n (%)	88 (31)	167 (24)§	86 (39)	254 (36)
HADS-Depression ≥ 10 points, n (%)	85 (30)	176 (25)	82 (38)	222 (31)
PImax, kPa	7.1 ± 2.1	7.5 ± 2.1*	5.9 ± 1.8	6.2 ± 1.9§
PImax, % predicted	68 ± 20	72 ± 19*	84 ± 25	88 ± 26§
PEmax, kPa	11.0 ± 3.7	11.8 ± 3.4*	8.5 ± 2.9	9.1 ± 3.0*
PEmax, % predicted	56 ± 19	60 ± 17*	63 ± 21	68 ± 22*
6MWD, m	386 ± 124	423 ± 112†	348 ± 116	395 ± 108†
6MWD, % predicted	58 ± 18	64 ± 16†	59 ± 18	66 ± 17†
CWRT time to exhaustion, s	304 ± 252	296 ± 207	225 ± 146	248 ± 165
1RM Leg press, kg	91 ± 44	101 ± 46*	53 ± 31	60 ± 30*
1RM Leg extension, kg	34 ± 13	38 ± 14†	21 ± 10	25 ± 10†

Abbreviations: BMI, body mass index; FFMI, Fat-Free Mass index; FEV_1_, forced expiratory volume in the first second; FVC, forced vital capacity; RV, residual volume; TLC, total lung capacity; TL_CO_, transfer capacity for carbon monoxide; LTOT, long-term oxygen therapy; GOLD, Global Initiative for Chronic Obstructive Lung Disease; mMRC, modified Medical Research Council; CAT, COPD Assessment Test; HADS, Hospital Anxiety and Depression Scale; PI_ma_x__, maximal inspiratory mouth pressure; PE_max_, maximal expiratory mouth pressure; 6MWD, 6-Minute Walk Distance; CWRT, constant work rate cycle test; 1RM, 1-repetition maximum.

† indicates a significant difference of *p* <0.001*.*

§ indicates a significant difference of *p* <0.05.

⁎ indicates a significant difference of *p* <0.01.

### Statistical analyses

All statistical analyses were performed using IBM SPSS Statistics 25 (SPSS Inc., Chicago, USA). Descriptive data were presented as mean ± SD unless stated otherwise. A priori, the level of significance was set at *p*<0.05.

Between-group differences were tested using an unpaired t-test for continuous variables and a chi-square test for categorical variables, as appropriate. Responsiveness of isokinetic quadriceps function to PR was tested using a paired sample t-test. MID estimates for isokinetic quadriceps function variables that were responsive to PR were determined using a combination of distribution-based and anchor-based techniques.^35^ Three distribution-based techniques were applied: standard error of measurement (SEM) = SD_baseline_ * $\sqrt{1-\text{intraclass correlation coefficient}}$; empirical rule effect size = 0.08 * 6 * SD_delta_; Cohen's effect size = 0.5 * SD_delta._^36^ Test-retest intraclass correlation coefficients were derived from previous studies in patients with COPD (ICCs: peak torque (Nm) = 0.97,^15^ total work (J) = 0.98,^15^^,^^16^ and work fatigue index_10_ (%) = 0.64–0.92).^15^ To perform the anchor-based method, a minimal correlation of 0.3 between the change in pre-determined anchors (CAT and 6MWD) and the change in muscle function was required to subsequently perform a linear regression and receiver operating characteristic (ROC) analysis.^35^^,^^37^ For the ROC analysis, an area under the curve of more than 0.7 was accepted as meaningful.^35^^,^^37^ Furthermore, the data were stratified for sex as several studies have reported the influence of sex on muscle function.^38^^,^^39^

## Results

### Characteristics

A flowchart illustrating how many patients performed the isokinetic quadriceps test and which criteria were assessed in terms of a correct performance at baseline is shown in Fig. 1. Pre-rehabilitation isokinetic muscle test data were available from 2033 of the 3152 patients and used for further analyses. These patients were 65±9 years old, had a BMI of 26±6 kg/m^2^, and forced expiratory volume in one second of 49±22% predicted (Table 2, Supplementary material).

**Table 2:** Changes in isokinetic quadriceps function after PR in male and female patients with COPD with a correct baseline and post PR isokinetic test.

	Male patients (*n* = 474)			Female patients (*n* = 513)		
	Baseline	Post PR	Delta	Baseline	Post PR	Delta
Peak torque (Nm)	112 ± 35	122 ± 36*^†^*	10 ± 13	73 ± 22	80 ± 22†	7 ± 9
Peak torque (% predicted)	67 ± 19	73 ± 19*^†^*	6 ± 8	64 ± 18	70 ± 18†	6 ± 8
Total work (J)	1947 ± 688	2210 ± 727*^†^*	263 ± 270	1283 ± 437	1481 ± 453†	198 ± 190
Work Fatigue index_10_ (%)	43 ± 14	43 ± 11	0 ± 12	45 ± 13	44 ± 10	−1 ± 12
Work Fatigue index_5_ (%)	49 ± 14	50 ± 13	1 ± 15	52 ± 14	51 ± 11	−1 ± 13

^§^indicates a significant difference between baseline and post PR of *p* <0.05; *indicates a significant difference between baseline and post PR of *p* <0.01

† indicates a significant difference between baseline and post PR of *p* <0.001. Abbreviations: COPD, chronic obstructive pulmonary disease; PR, pulmonary rehabilitation.

### Test performance criteria pre PR

Of all 2033 patients who performed the isokinetic quadriceps test at baseline and had complete data available, 193 patients (10%) were not able to complete all 30 repetitions due to various reasons (i.e. dyspnea, fatigue; Fig. 1). The second criterion, to reach the maximal torque value within the first five repetitions, was not observed in 328 patients (16%). Seventy percent of these patients obtained their peak torque between repetition 6 and 10, while 5% produced their peak torque between repetition 20 and 30. The third criterion, work fatigue (less work in the last 10 repetitions compared to the first 10 repetitions), was not present in 18 patients (1%). Thus, in total 539 patients (27%) did not fulfill at least one of three test performance criteria (Fig. 1).

Male and female patients who incorrectly performed the isokinetic quadriceps test at baseline had a significantly lower exercise capacity, and a lower isotonic peripheral and respiratory muscle strength than male and female patients who performed the test correctly (all *p*-values <0.05). In males, an incorrect measurement was also related to higher dyspnea severity and anxiety scores. Age, pulmonary function, body composition, and disease severity (GOLD) did not differ between patients who correctly and incorrectly performed the isokinetic quadriceps test (Table 1).

### Test performance criteria post PR

**Fig. 2:**
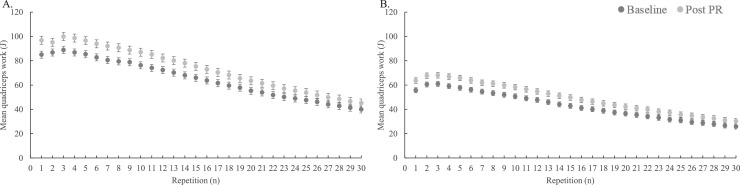
Baseline and post pulmonary rehabilitation (PR) mean quadriceps work (J) per repetition with standard error for male (A) and female (B) patients with chronic obstructive pulmonary disease.

Of the 1494 patients who performed the baseline isokinetic quadriceps test correctly, 1106 patients also had complete post PR data. 35 patients (3%) did not complete all 30 repetitions post PR; 82 patients (8%) did not obtain a peak torque within the first five repetitions; work fatigue was not present in two patients (0.2%). Thus, a total of 987 patients (89%) performed the post PR assessment correctly (Fig. 2, Supplementary material).

### Responsiveness and MID estimates

Responsiveness and MID estimates were determined for male and female patients with a correct baseline and post PR isokinetic test performance (*n*=987). Baseline characteristics for these patients are illustrated in the supplementary material (Table 3, Supplementary material). Following PR, male and female patients improved their isokinetic quadriceps peak torque by 10±13 Nm and 7±9 Nm and total work by 263±270 J and 198±190 J, respectively (all *p*-values <0.05). Both work fatigue indexes did not change after PR (Table 2). The mean quadriceps work in J per repetition of the baseline and post PR isokinetic tests for males and females are depicted in Fig. 2, demonstrating an overall increase in mean work per repetition following PR. In addition, CAT and 6MWD improved significantly in male and female patients (∆CAT= −3±6 points and −4±6 points, ∆6MWD=19±62 m and 19±57 m, respectively; all *p*-values <0.001).

**Table 3:** Distribution-based estimates of the minimal important difference (MID) in isokinetic quadriceps function for male and female patients with COPD.

	Males (*n* = 474)				Females (*n* = 513)			
Method	SEM	Empirical rule effect size	Cohen's effect size	MID estimates	SEM	Empirical rule effect size	Cohen's effect size	MID estimates
Peak torque (Nm)	6	6	7	6–7	4	4	5	4–5
Peak torque (% change)	NA	8	8	8	NA	8	8	8
Total work (J)	97	129	135	97–135	62	95	99	62–99
Total work (% change)	NA	24	25	24–25	NA	13	14	13–14

Abbreviations: COPD, chronic obstructive pulmonary disease; NA, not applicable; SEM, standard error of measurement.

MID estimates were calculated for peak torque and total work, both in absolute values and as percentage change from baseline, as these outcomes turned out to be responsive to PR. Unfortunately, MID estimates could not be calculated using both anchor-based methods (linear regression and ROC analyses) as all correlation coefficients between changes in isokinetic function and changes in 6MWD and CAT score were <0.3 (Table 4, Supplementary material). Using distribution-based calculations, MID estimates for peak torque ranged between 6 and 7 Nm or 8% change in males and 4–5 Nm or 8% change in females. For total work, MIDs ranged between 97 and 135 J or 24–25% change in males and between 62–99 J or 13–14% change in females (Table 3).

### Isokinetic test performance criteria

The two most prevalent reasons for an incorrect isokinetic test performance in patients with COPD were a peak torque between repetition 6 and 10 (PT_6-10_, *n*=229) and premature test termination between repetition 20 and 29 (REP_20-29_, *n*=103). At baseline, patients with PT_6-10_ were characterized with lower peak torque and smaller work fatigue index (all *p*-values <0.05) compared to patients with a correct test performance. Patients with REP_20-29_ reported a lower peak torque percentage predicted and smaller total work and work fatigue index_5_ than patients with a correct test performance (all *p*-values <0.05) (Table 5, Supplementary material). Furthermore, the responsiveness to PR was different between patients with PT_6-10_ and REP_20-29_ compared to patients with a correct test performance. Both groups reported a significant increase in work fatigue index_5_ (PT_6-10_: 4±23 % and REP_20-29_: 13±36 %, both *p*-values <0.05), while patients with a correct test performance had no change following PR. In addition, peak torque as percentage predicted improved more in patients with PT_6-10_ (8±9 %) than in patients with a correct test performance (6±8 %) (Table 6, Supplementary material).

## Discussion

The present study is the first to provide an extensive overview of the rate of patients with positive test performance criteria, responsiveness, and MID estimates of isokinetic evaluation of quadriceps function in male and female patients with COPD following PR. At baseline, one in four patients was not able to perform the test correctly according to the pre-defined test criteria. Generally, quadriceps peak torque (strength) and total work (endurance) improved in male and female patients following PR. The MID estimates were 6–7 Nm and 97–135 J for males and 4–5 Nm and 62–99 J for females, respectively.

### Rate of patients with positive test performance criteria

Even though Frykholm et al.^16^ reported great feasibility of the isokinetic quadriceps test in terms of test duration, the current study found that one in four patients in a clinical setting was not able to perform the test correctly. The two most common reasons were premature test termination between repetition 20 and 29 (instead of 30), and peak torque reached between repetition 6 and 10 (instead of within the first five repetitions). These male and female patients with an incorrect test performance at baseline were weaker and had a lower exercise capacity. In addition, patients with an incorrect test performance report lower values for total work and work fatigue index, which is expected to reflect the difference in test performance rather than a reduced quadriceps endurance. A familiarization session may increase the rate of patients with a correct test performance based on the pre-defined criteria.^15^^,^^16^ However, due to time and personnel constraints, this will not always be possible in clinical practice.

### Responsiveness

In patients with COPD and a correct baseline and post PR isokinetic test, peak torque and total work were responsive following PR as they improved with 9% and 14% in males and 10% and 15% in females, respectively. The improvement in peak torque is in accordance with prior studies.^40^, ^41^, ^42^, ^43^ In addition, there is a moderate-to-high correlation between the change in peak torque and total work (*r*= 0.723, *p*<0.001), highlighting the influence of muscle strength on total work. Previous literature regarding the responsiveness of isokinetic quadriceps endurance identified positive effects of different exercise interventions on quadriceps endurance in patients with COPD.^44^ However, these studies used different testing protocols, interventions, or outcome measures.^44^ This study did not find an improvement in work fatigue index following PR. The exercise-based interventions of the current PR program did not specifically focus on the fatigability of the quadriceps, which may explain the lack of change. Therefore, it is recommended to monitor the training intervention more extensively in future research.

### MID estimates

The current study shows that MIDs for males and females were different. Therefore, sex-specific MIDs are recommended to be applied to obtain a more accurate interpretation of the efficacy of specific interventions, and can be used to determine the ‘number needed to treat’ for future intervention studies.^45^ It is important to note that the MID estimates only apply for patients with a correct baseline and post PR isokinetic test. The changes in the 6MWD and the CAT showed only weak correlations with the changes in peak torque and total work. Whether changes in other outcomes (such as the Short Physical Performance Battery or the endurance shuttle walk test) can be used as anchors, remains to be determined.

### Methodological limitations

The high correlation between peak torque and total work reflects the influence of muscle strength on total work, which is expected based on its formula ‘work = force*distance’. This raises the question of whether total work is an appropriate outcome measure of quadriceps endurance. In addition, weak and similar correlations were seen between peak torque (strength) and total work (endurance) with 6MWD. This might indicate that the applied outcome measures for muscle strength and endurance partly represent associated aspects of muscle function. However, recent studies have demonstrated that peak torque and total work are two different aspects of quadriceps function which independently correlate to exercise capacity.^10^^,^^46^ Future research should determine the optimal protocol and outcome measures for evaluating quadriceps endurance. For now, it is important to make a clear distinction between quadriceps endurance and total work and not use both terms interchangeably.

In addition, the exercise training is not identical for all patients due to the patient-tailored nature of the PR program. Unfortunately, we were not able to retain the performed exercise training parameters per individual due to the retrospective design of this study. In general, the main goal of the prescribed resistance training in this study was to improve muscle strength rather than muscle endurance. Therefore, potentially greater improvements in quadriceps muscle endurance could be achieved if combining aerobic training^1^ with low-load/high-repetition resistance training.^47^ For future studies, it is recommended to extensively monitor and report the training parameters.

Finally, data were obtained from one location and included only a selected group of patients with COPD, specifically those who were more severe and dyspnoeic. This should be noted when interpreting the results as it reduces generalizability.

### Clinical implications

Based on the results of this study, we recommend using the pre-defined test criteria for isokinetic test performance in clinical practice. Furthermore, the test can be used to evaluate PR efficacy on peak torque and total work in individual patients with COPD. Whether this isokinetic protocol is suitable to determine changes in work fatigue index following PR has to be investigated in future studies using exercise interventions focussing on improving fatigability.

We are aware that a computerized dynamometer is a complex and expensive equipment and is not available in all settings. However, this study is relevant for the centres that already use a computerized dynamometer or have the opportunity to purchase one. Furthermore, it is of great importance to create a standardized operating procedure for isokinetic quadriceps testing and determine reference values regarding quadriceps endurance.

## Conclusions

Based on the current test criteria, three in four patients with COPD performed the isokinetic quadriceps test correctly during baseline PR assessment. These patients were stronger and had a higher exercise capacity than patients with an incorrect test performance. Furthermore, peak torque and total work, but not work fatigue index, were responsive to PR and sex-specific MIDs were established. However, future studies are needed to determine prediction equations and/or normal values to improve the interpretation of isokinetic quadriceps endurance.

## Conflict of interest

The BASES consortium is financially supported by Lung Foundation, the Netherlands [#5.1.18.232]. Drs. Jana De Brandt is funded by the Flemish government. The Research of FWO Aspirant Jana De Brandt is sponsored by FWO-grant [#11B4718N]. Dr. F.M.E. Franssen is supported by grants and personal fees from AstraZeneca, personal fees from Boehringer Ingelheim, personal fees from Chiesi, personal fees from GlaxoSmithKline, grants and personal fees from Novartis, personal fees from TEVA, outside the submitted work. Dr. B. van den Borst is supported by personal lecture fees from AstraZeneca and Boehringer Ingelheim bv. Drs. A.A.F. Stoffels, R. Meys, Dr. H.W.H. van Hees, A.J. van ‘t Hul, P.H. Klijn, A.W. Vaes, J. De Brandt and Prof. Dr. C. Burtin and M.A. Spruit declare that they do not have a conflict of interest

## Appendix Supplementary materials

Image, application 1

## Footnotes

Supplementary material associated with this article can be found in the online version at doi:10.1016/j.bjpt.2022.100451.
