# Supplementary material for: Isokinetic testing of quadriceps function in COPD: feasibility, responsiveness, and minimal important differences in patients undergoing pulmonary rehabilitation
Source: Braz J Phys Ther. 2022 Oct 17;26(5):100451. doi: 10.1016/j.bjpt.2022.100451 (PMC9593178; doi:10.1016/j.bjpt.2022.100451)
Supplement: Supplementary file 1 [file mmc1.docx]

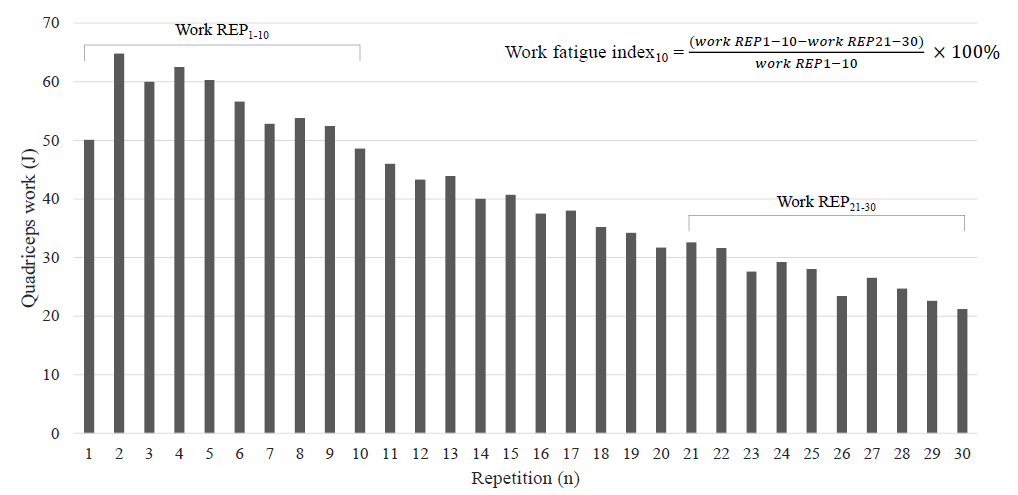


**Supplementary material - Figure 1.** Quadriceps work (J) per repetition of one patient to visualize the work fatigue index_10_.

*Abbreviations: REP, repetition.*

**Isokinetic quadriceps measurements not performed during post PR assessment (n = 370)**

- No post PR assessment performed: 287
- Medical reason(s): 51
  - Passed away: 1
  - Problems with musculoskeletal system: 39
  - Hospital admission: 2
  - Exacerbation: 1
  - Other medical reason: 8
- Logistic reason(s): 23
- Reason unknown/not filled in by technician: 6
- Motivational problem(s): 3

**Isokinetic quadriceps measurements correctly performed during BA**

(n = 1494)

**Isokinetic quadriceps measurements performed**

(n = 1124)

**No complete data available regarding isokinetic measurement (n = 18)**

**Less than 30 repetitions completed (n = 35)***

- < 10 repetitions: 1
  - Fatigue: 1
- 10-19 repetitions: 11
  - Musculoskeletal problems: 7
  - Fatigue: 3
  - Dyspnea: 1
- 20-29 repetitions: 23
  - Musculoskeletal problems: 10
  - Fatigue: 6
  - Dyspnea: 6
  - Not mentioned: 3

**Complete data available**

(n = 1106)

**30 repetitions completed**

(n = 1071)

AND

**Presence of work fatigue**

(n = 987)

**Peak torque rep in first 5 repetitions**

(n = 989)

**Peak torque rep *not* in first 5 repetitions (n = 82)****

- Peak torque *rep* between 6-10 repetitions: 70
- Peak torque *rep* between 11-20 repetition: 12

AND

**No presence of work fatigue (n = 2)*****

**Correctly performed isokinetic quadriceps measurements**

(n = 987)

**Supplementary material - Figure 2.** Flowchart of patients that performed the post PR isokinetic quadriceps measurement correctly according to the following three criteria: 30 repetitions completed, peak torque within first 5 repetitions and presence of work fatigue.

*Abbreviations: BA, baseline assessment; PR, pulmonary rehabilitation; rep, repetition.. *Some patients reported multiple reasons for early test termination. **5 patients with the highest peak torque not in the first 5 repetitions were already excluded due to the fact that they performed less than 30 repetitions. ***7 patients with no presence of work fatigue were already excluded due to the fact that their peak torque was not in the first 5 repetitions.*

**Supplementary material – Table 1.** Training parameters for the different types of cycling and walking training.

| **Modality** | **Type** | **Intensity** | **Duration** | **Rest** | **Progression** |
| --- | --- | --- | --- | --- | --- |
| Cycling | Endurance/recovery | 40% Wmax | 1 x 20 min |  | 5% each 2 weeks |
| Cycling | Extensive interval | 60% Wmax | 8 x 2 min | 1 min | 5% each week |
| Cycling | Intensive interval | 80% Wmax | 12 x 1 min | 1 min | 10% each 2 weeks |
| Walking | Endurance/recovery | 60% 6MWD | 1 x 20 min |  | 5% each week |
| Walking | Extensive interval | 75% 6MWD | 4 x 5 min | 1 min | 10% each week (first 4 weeks), 10% each 2 weeks (second 4 weeks) |
| Walking | Intensive interval | 100% 6MWD | 12 x 1 min | 1 min | 10% each 2 weeks |

*Abbreviations: Wmax, maximal workload; 6MWD, 6-Minute Walk Distance.*

|  | Mean ± SD | N |
| --- | --- | --- |
| Age, years | 65 ± 9 | 2033 |
| BMI, kg/m^2^ | 26.2 ± 6.0 | 2033 |
| FFMI, kg/m^2^ | 16.6 ± 2.5 | 1993 |
| FEV_1_, L | 1.28 ± 0.66 | 2030 |
| FEV_1_, % predicted | 49 ± 22 | 2030 |
| FEV_1_/FVC, % | 40 ± 14 | 2030 |
| RV/TLC, % | 52 ± 12 | 1964 |
| TL_CO_, % predicted | 50 ± 17 | 1885 |
| Smoking, packs per year | 44 ± 24 | 1891 |
| LTOT, n (%) | 404 (20) | 1988 |
| GOLD (1/2/3/4), % | 10/31/37/22 | 2030 |
| GOLD (A/B/C/D), % | 8/24/8/60 | 2011 |
| mMRC ≥ 2, n (%) | 1706 (85) | 2014 |
| CAT total ≥ 18, n (%) | 1420 (74) | 1927 |
| HADS-Anxiety ≥ 10, n (%) | 595 (31) | 1919 |
| HADS-Depression ≥ 10, n (%) | 565 (29) | 1919 |
| PImax, kPa | 6.8 ± 2.1 | 2012 |
| PImax, % predicted | 79 ± 24 | 2008 |
| PEmax, kPa | 10.3 ± 3.5 | 1865 |
| PEmax, % predicted | 63 ± 20 | 1861 |
| 6MWD, m | 399 ± 115 | 2019 |
| CWRT time to exhaustion, s | 272 ± 196 | 1851 |
| 1RM Leg press, kg | 79 ± 44 | 1973 |
| 1RM Leg extension, kg | 31 ± 14 | 1942 |

**Supplementary material - Table 2.** Baseline characteristics of all patients with complete data available regarding isokinetic testing.

*Abbreviations: BMI, body mass index; FFMI, Fat-Free Mass index; FEV_1_, forced expiratory volume in the first second; FVC, forced vital capacity; RV, residual volume; TLC, total lung capacity; TL_CO_, transfer capacity for carbon monoxide; LTOT, long-term oxygen therapy; GOLD, Global Initiative for Chronic Obstructive Lung Disease; mMRC, modified Medical Research Council; CAT, COPD Assessment Test; HADS, Hospital Anxiety and Depression Scale; PImax, maximal inspiratory mouth pressure; PEmax, maximal expiratory mouth pressure; 6MWD, 6-Minute Walk Distance; CWRT, constant work rate cycle test; 1RM, 1-repetition maximum.*

**Supplementary material - Table 3.** Baseline characteristics of male and female patients with COPD with a correct baseline and post PR isokinetic test performance.

|  | Male patients (n = 474) | | Female patients (n = 513) | |
| --- | --- | --- | --- | --- |
|  | **Mean ± SD** | **N** | **Mean ± SD** | **N** |
| Age, years | 67 ± 9 | 474 | 63 ± 8 | 513 |
| BMI, kg/m^2^ | 26 ± 5 | 474 | 26 ± 6 | 513 |
| FFMI, kg/m^2^ | 18 ± 2 | 467 | 15 ± 2 | 506 |
| FEV_1_, L | 1.50 ± 0.71 | 473 | 1.06 ± 0.49 | 513 |
| FEV_1_, % predicted | 50 ± 22 | 473 | 48 ± 20 | 513 |
| FEV_1_/FVC, % | 39 ± 15 | 473 | 29 ± 13 | 513 |
| RV/TLC, % | 49 ± 11 | 465 | 55 ± 11 | 496 |
| T_LCO_, % predicted | 52 ± 18 | 461 | 48 ± 15 | 475 |
| Smoking, packs per year | 45 ± 24 | 437 | 41 ± 23 | 495 |
| LTOT, n (%) | 82 (18) | 468 | 115 (23) | 501 |
| GOLD (1/2/3/4), % | 11/30/39/20 | 473 | 8/30/42/20 | 513 |
| GOLD (A/B/C/D), % | 12/24/10/54 | 467 | 6/25/7/62 | 509 |
| mMRC ≥ 2, n (%) | 367 (79) | 467 | 444 (87) | 510 |
| CAT total ≥ 18, n (%) | 315 (70) | 448 | 386 (78) | 493 |
| HADS-Anxiety ≥ 10, n (%) | 98 (22) | 448 | 166 (34) | 493 |
| HADS-Depression ≥ 10, n (%) | 111 (25) | 448 | 155 (31) | 493 |
| PImax, kPa | 7.6 ± 2.1 | 471 | 6.4 ± 1.9 | 510 |
| PImax, % predicted | 73 ± 19 | 471 | 91 ± 26 | 509 |
| PEmax, kPa | 11.8 ± 3.3 | 425 | 9.4 ± 3.0 | 491 |
| PEmax, % predicted | 61± 17 | 425 | 70 ± 22 | 490 |
| 6MWD, m | 435 ± 111 | 470 | 401 ± 108 | 512 |
| CWRT time, s | 302 ± 206 | 453 | 253 ± 167 | 484 |
| 1RM Leg press, kg | 103 ± 45 | 462 | 62 ± 31 | 501 |
| 1RM Leg extension, kg | 39 ± 14 | 453 | 25 ± 10 | 500 |

*Abbreviations: BMI, body mass index; FFMI, Fat-Free Mass index; FEV_1_, forced expiratory volume in the first second; FVC, forced vital capacity; RV, residual volume; TLC, total lung capacity; T_LCO_, transfer capacity for carbon monoxide; LTOT, long-term oxygen therapy; GOLD, Global Initiative for Chronic Obstructive Lung Disease; mMRC, modified Medical Research Council; CAT, COPD Assessment Test; HADS, Hospital Anxiety and Depression Scale; PIMAX, maximal inspiratory mouth pressure; PEMAX, maximal expiratory mouth pressure; 6MWD, 6-Minute Walk Distance; CWRT, constant work rate cycle test; 1RM, 1-repetition maximum.*

**Supplementary material - Table 4.** Pearson correlations between change in isokinetic quadriceps function and change in anchors 6MWD and CAT in male and female patients with COPD.

|  | **Male patients (n = 474)** | | | | **Female patients (n = 513)** | | | |
| --- | --- | --- | --- | --- | --- | --- | --- | --- |
|  | **∆6MWD** | | **∆CAT** | | **∆6MWD** | | **∆CAT** | |
|  | **R** | **N** | **R** | **N** | **R** | **N** | **R** | **N** |
| ∆Peak torque (Nm) | 0.151* | 466 | -0.073 | 423 | 0.171* | 507 | -0.101* | 478 |
| ∆Peak torque (% change) | 0.189* | 466 | -0.082 | 423 | 0.197* | 507 | -0.076 | 478 |
| ∆Total work (J) | 0.193* | 466 | 0.015 | 423 | 0.227* | 507 | -0.159* | 478 |
| ∆Total work (% change) | 0.196* | 466 | -0.076 | 423 | 0.233* | 507 | -0.096* | 478 |

*Abbreviations: 6MWD, 6-Minute Walk Distance; CAT, COPD Assessment Test. * indicates a p <0.05.*

**Supplementary material - Table 5.** Baseline isokinetic quadriceps function of patients with an incorrect isokinetic test performance (based solely on peak torque between repetition 6 and 10 and premature test termination between repetition 20 and 29) and the reference group of patients with a correct baseline isokinetic test performance.

|  | **Reference group**  **(n = 1494)** | **Peak torque between**  **repetition 6 – 10**  **(n = 217)** | **Number of repetitions**  **between 20 – 29**  **(n = 83)** |
| --- | --- | --- | --- |
| Peak torque (Nm) | 90 ± 35 | 83 ± 33* | 87 ± 35 |
| Peak torque (% predicted) | 64 ± 19 | 58 ± 18** | 58 ± 19* |
| Total work (J) | 1573 ± 669 | 1513 ± 682 | 1206 ± 536** |
| Work Fatigue index_10_ (%) | 44 ± 13 | 32 ± 15** | 39 ± 48 |
| Work Fatigue index_5_ (%) | 50 ± 15 | 30 ± 21** | 45 ± 20* |

** indicates a significant difference of p<0.05, ** indicates a significant difference of p<0.001.*

**Supplementary material - Table 6.** Baseline, post PR and delta (post PR – baseline) isokinetic quadriceps function of patients with an incorrect isokinetic test performance at baseline and/or post PR (based solely on peak torque between repetition 6 and 10 and premature test termination between repetition 20 and 29) and the reference group of patients with a correct baseline and post PR isokinetic test performance.

|  | **Reference group**  **(n = 987)** | | | | **Peak torque between repetition**  **6 – 10 (n = 211)** | | | | **Number of repetitions**  **between 20 – 29 (n = 74)** | | | |
| --- | --- | --- | --- | --- | --- | --- | --- | --- | --- | --- | --- | --- |
|  | **Baseline** | **Post PR** | **Delta** | **Pre** | | **Post PR** | **Delta** | **Pre** | | **Post PR** | **Delta** |  |
| Peak torque (Nm) | 91 ± 35 | 100 ± 36** | 8 ± 12 | 88 ± 34 | | 99 ± 35** | 10 ± 14 | 89 ± 35 | | 99 ± 35** | 10 ± 15 |  |
| Peak torque (% predicted) | 65 ± 19 | 72 ± 19** | 6 ± 8 | 60 ± 18 | | 68 ± 18** | 8 ± 9^$^ | 59 ± 18 | | 66 ± 16** | 7 ± 10 |  |
| Total work (J) | 1602 ± 661 | 1831 ± 702** | 229 ± 234 | 1607 ± 669 | | 1870 ± 686** | 263 ± 265 | 1376 ± 553 | | 1482 ± 640 | 106 ± 904 |  |
| Work Fatigue index_10_ (%) | 44 ± 13 | 44 ± 11 | 0 ± 12 | 34 ± 15 | | 34 ± 14 | 0 ± 16 | 51 ± 35 | | 51 ± 25 | 0 ± 47 |  |
| Work Fatigue index_5_ (%) | 50 ± 15 | 50 ± 12 | 0 ± 14 | 35 ± 20 | | 39 ± 17* | 4 ± 23^$^ | 46 ± 18 | | 59 ± 26* | 13 ± 36^$^ |  |

** indicates a significant difference between baseline and post PR of p<0.05, ** indicates a significant difference between baseline and post PR of p<0.001, ^$^ indicates a significant difference between deltas in comparison to the reference group of p<0.05, ^$$^ indicates a significant difference between deltas in comparison to the reference group of p<0.001.*
